# (1*S*,2*S*,4*R*)-7-*tert*-But­oxy­bicyclo­[2.2.1]hept-5-en-2-yl (2*S*)-2-(6-meth­oxy­naphthalen-2-yl)propano­ate

**DOI:** 10.1107/S1600536811024238

**Published:** 2011-06-30

**Authors:** Alan J. Lough, David A. Petrone, William Tam

**Affiliations:** a Department of Chemistry, University of Toronto, Toronto, Ontario, Canada M5S 3H6; b Department of Chemistry, University of Guelph, Guelph, Ontario, Canada N1G 2W1

## Abstract

In the title mol­ecule, C_25_H_30_O_4_, the napthalene ring system is slightly bowed, with a dihedral angle of 4.37 (13)° between the two benzene rings.

## Related literature

For the synthesis of *anti*-2,7-disubstituted norbornadienes from racemic 7-*tert*-but­oxy-bicyclo­[2.2.1]hepta-5-en-2-ol, see: Tsui *et al.* (2009 ▶).
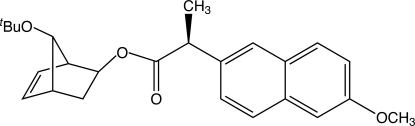


## Experimental

### 

#### Crystal data

C_25_H_30_O_4_
*M* *_r_* = 394.49
Monoclinic, 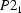

*a* = 11.8328 (11) Å
*b* = 6.0084 (4) Å
*c* = 15.2205 (15) Å
β = 96.705 (4)°
*V* = 1074.72 (16) Å^3^
*Z* = 2
Mo *K*α radiation
μ = 0.08 mm^−1^
*T* = 150 K
0.50 × 0.20 × 0.12 mm

#### Data collection

Nonius KappaCCD diffractometer
Absorption correction: multi-scan (*SORTAV*; Blessing, 1995 ▶) *T*_min_ = 0.480, *T*_max_ = 0.990
8333 measured reflections
2649 independent reflections
2023 reflections with *I* > 2σ(*I*)
*R*_int_ = 0.071

#### Refinement

*R*[*F* ^2^ > 2σ(*F* ^2^)] = 0.048
*wR*(*F* ^2^) = 0.122
*S* = 1.05
2649 reflections
267 parameters
1 restraint
H-atom parameters constrained
Δρ_max_ = 0.16 e Å^−3^
Δρ_min_ = −0.25 e Å^−3^

### 

Data collection: *COLLECT* (Nonius, 2002 ▶); cell refinement: *DENZO-SMN* (Otwinowski & Minor, 1997 ▶); data reduction: *DENZO-SMN*; program(s) used to solve structure: *SIR92* (Altomare *et al.*, 1994 ▶); program(s) used to refine structure: *SHELXTL* (Sheldrick, 2008 ▶); molecular graphics: *PLATON* (Spek, 2009 ▶); software used to prepare material for publication: *SHELXTL*.

## Supplementary Material

Crystal structure: contains datablock(s) global, I. DOI: 10.1107/S1600536811024238/pk2333sup1.cif

Structure factors: contains datablock(s) I. DOI: 10.1107/S1600536811024238/pk2333Isup2.hkl

Supplementary material file. DOI: 10.1107/S1600536811024238/pk2333Isup3.cml

Additional supplementary materials:  crystallographic information; 3D view; checkCIF report

## Figures and Tables

**Fig. 1.**
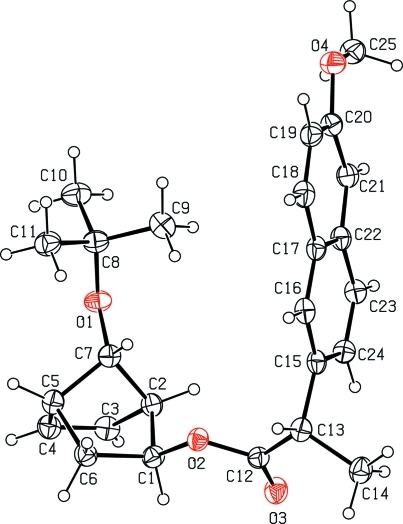
The molecular structure of the title compound with displacement ellipsoids drawn at the 30% probabilty level.

**Fig. 2.**
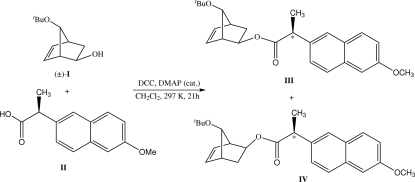
The reaction scheme

## supplementary crystallographic information

### Comment

Recently, we have investigated the synthesis of anti-2,7-disubstituted norbornadienes from racemic 7-*tert*-butoxy-bicyclo[2.2.1]hepta-5-en-2-ol (±) (I) (Tsui *et al.*, 2009). In order to synthesize a chiral anti-2,7-disubstituted norbornadiene, we have studied the resolution of the racemic mixture of 7-*tert*-butoxy-bicyclo[2.2.1]hepta-5-en-2-ol (±) (I) through the use of α-chiral carboxylic acids as resolving agents. We found that when (*S*)-(+)-6-methoxy-α-methyl-2-naphthaleneacetic acid (II) (See Fig. 2) is used as the resolving agent, the diastereomeric mixture of (III) and (IV) is obtained. This mixture has been resolved using fractional crystallization which afforded diastereomer (IV) with dr > 99:1. The absolute position of the ester on the bicyclic framework has been elucidated by this single-crystal *x*-ray analysis.

### Experimental

7-*tert*-butoxy-bicyclo[2.2.1]hepta-5-en-2-ol (±)-I (1.2 g, 6.4 mmol), (*S*)-(+)-6-methoxy-a-methyl-2-napthaleneacetic acid (II) (1.63 g, 7.08 mmol), DCC (1.58 g, 7.68 mmol), and DMAP (0.156 g, 1.28 mmol) were weighted into a dry round bottom flask and purged with nitrogen. Dried CH_2_Cl_2_ (64 ml) was added through a vented septum to dissolve all solids forming a 0.1 *M* solution with respect to (±)-I. The reaction vessel was sealed and its contents were stirred at room temperature for 21 h while monitoring the reaction progression by TLC. The crude product was purified using flash column chromatography (EtOAc:hexanes = 30:70) to provide diastereomers (III) and (IV) in equal ratio as an off white semi-solid (2.33 g, 5.9 mmol, 92%). Fractional crystallization with MeOH was used to separate these species and to grow suitable crystals of (IV).

### Refinement

H atoms were placed in calculated positions with C—H = 0.95–0.99 Å and included in a riding-motion approximation with *U*_iso_(H) = 1.2*U*_eq_(C) or 1.2*U*_eq_(C_methyl_). In the absence of significant anomalous dispersion effects the Friedel pairs were merged before refinement. The absolute stereochemistry was determined by an unchanging chiral center in the synthesis.

### Crystal data

**Table:**

C_25_H_30_O_4_	*F*(000) = 424
*M**_r_* = 394.49	*D*_x_ = 1.219 Mg m^−^^3^
Monoclinic, *P*2_1_	Mo *K*α radiation, λ = 0.71073 Å
Hall symbol: P 2yb	Cell parameters from 8333 reflections
*a* = 11.8328 (11) Å	θ = 2.7–27.5°
*b* = 6.0084 (4) Å	µ = 0.08 mm^−^^1^
*c* = 15.2205 (15) Å	*T* = 150 K
β = 96.705 (4)°	Needle, colourless
*V* = 1074.72 (16) Å^3^	0.50 × 0.20 × 0.12 mm
*Z* = 2	

### Data collection

**Table:**

Nonius KappaCCD diffractometer	2649 independent reflections
Radiation source: fine-focus sealed tube	2023 reflections with *I* > 2σ(*I*)
graphite	*R*_int_ = 0.071
Detector resolution: 9 pixels mm^-1^	θ_max_ = 27.5°, θ_min_ = 2.7°
φ scans and ω scans with κ offsets	*h* = −15→15
Absorption correction: multi-scan (*SORTAV*; Blessing, 1995)	*k* = −7→6
*T*_min_ = 0.480, *T*_max_ = 0.990	*l* = −16→19
8333 measured reflections	

### Refinement

**Table:**

Refinement on *F*^2^	Primary atom site location: structure-invariant direct methods
Least-squares matrix: full	Secondary atom site location: difference Fourier map
*R*[*F*^2^ > 2σ(*F*^2^)] = 0.048	Hydrogen site location: inferred from neighbouring sites
*wR*(*F*^2^) = 0.122	H-atom parameters constrained
*S* = 1.05	*w* = 1/[σ^2^(*F*_o_^2^) + (0.0605*P*)^2^ + 0.0914*P*] where *P* = (*F*_o_^2^ + 2*F*_c_^2^)/3
2649 reflections	(Δ/σ)_max_ < 0.001
267 parameters	Δρ_max_ = 0.16 e Å^−^^3^
1 restraint	Δρ_min_ = −0.25 e Å^−^^3^

### Special details

**Table:**

Experimental. multi-scan from symmetry-related measurements (SORTAV (Blessing 1995)
Geometry. All e.s.d.'s (except the e.s.d. in the dihedral angle between two l.s. planes) are estimated using the full covariance matrix. The cell e.s.d.'s are taken into account individually in the estimation of e.s.d.'s in distances, angles and torsion angles; correlations between e.s.d.'s in cell parameters are only used when they are defined by crystal symmetry. An approximate (isotropic) treatment of cell e.s.d.'s is used for estimating e.s.d.'s involving l.s. planes.
Refinement. Refinement of *F*^2^ against ALL reflections. The weighted *R*-factor *wR* and goodness of fit *S* are based on *F*^2^, conventional *R*-factors *R* are based on *F*, with *F* set to zero for negative *F*^2^. The threshold expression of *F*^2^ > σ(*F*^2^) is used only for calculating *R*-factors(gt) *etc*. and is not relevant to the choice of reflections for refinement. *R*-factors based on *F*^2^ are statistically about twice as large as those based on *F*, and *R*- factors based on ALL data will be even larger.

### Fractional atomic coordinates and isotropic or equivalent isotropic displacement parameters (Å^2^)

**Table:**

	*x*	*y*	*z*	*U*_iso_*/*U*_eq_	
O1	0.69010 (15)	0.8083 (4)	0.08425 (13)	0.0433 (5)	
O2	0.58736 (15)	0.8603 (3)	0.34025 (12)	0.0371 (4)	
O3	0.49435 (16)	0.5653 (4)	0.38828 (14)	0.0478 (5)	
O4	1.21074 (16)	0.2459 (4)	0.25951 (14)	0.0490 (5)	
C1	0.5058 (2)	0.8713 (5)	0.26074 (17)	0.0378 (6)	
H1A	0.4278	0.8258	0.2729	0.045*	
C2	0.5477 (2)	0.7305 (5)	0.18658 (18)	0.0368 (6)	
H2A	0.5760	0.5779	0.2037	0.044*	
C3	0.4558 (2)	0.7435 (5)	0.10890 (19)	0.0430 (7)	
H3	0.4070	0.6258	0.0866	0.052*	
C4	0.4559 (2)	0.9493 (5)	0.0778 (2)	0.0430 (7)	
H4	0.4061	1.0059	0.0295	0.052*	
C5	0.5482 (2)	1.0785 (5)	0.13189 (18)	0.0406 (7)	
H5A	0.5767	1.2140	0.1033	0.049*	
C6	0.5073 (2)	1.1131 (5)	0.2247 (2)	0.0407 (7)	
H6A	0.5610	1.2079	0.2629	0.049*	
H6B	0.4306	1.1809	0.2196	0.049*	
C7	0.6362 (2)	0.8921 (5)	0.15578 (18)	0.0377 (6)	
H7A	0.6934	0.9373	0.2062	0.045*	
C8	0.8098 (2)	0.8585 (6)	0.0853 (2)	0.0432 (7)	
C9	0.8780 (3)	0.7327 (6)	0.1619 (2)	0.0514 (8)	
H9A	0.8590	0.5740	0.1578	0.077*	
H9B	0.8589	0.7916	0.2184	0.077*	
H9C	0.9596	0.7524	0.1585	0.077*	
C10	0.8387 (3)	0.7701 (6)	−0.0023 (2)	0.0559 (9)	
H10A	0.8232	0.6099	−0.0059	0.084*	
H10B	0.9194	0.7965	−0.0072	0.084*	
H10C	0.7922	0.8465	−0.0507	0.084*	
C11	0.8306 (3)	1.1075 (6)	0.0922 (2)	0.0506 (8)	
H11A	0.7856	1.1829	0.0428	0.076*	
H11B	0.9116	1.1379	0.0900	0.076*	
H11C	0.8082	1.1624	0.1483	0.076*	
C12	0.5739 (2)	0.6939 (5)	0.39715 (18)	0.0364 (6)	
C13	0.6718 (2)	0.6822 (5)	0.46982 (18)	0.0374 (6)	
H13A	0.6969	0.8371	0.4863	0.045*	
C14	0.6351 (3)	0.5668 (6)	0.55187 (19)	0.0474 (7)	
H14A	0.5719	0.6494	0.5727	0.071*	
H14B	0.6993	0.5629	0.5987	0.071*	
H14C	0.6105	0.4145	0.5366	0.071*	
C15	0.7694 (2)	0.5613 (5)	0.43366 (17)	0.0359 (6)	
C16	0.8758 (2)	0.6525 (5)	0.43437 (18)	0.0384 (6)	
H16A	0.8912	0.7933	0.4616	0.046*	
C17	0.9630 (2)	0.5422 (5)	0.39564 (18)	0.0376 (6)	
C18	1.0740 (2)	0.6343 (5)	0.3944 (2)	0.0437 (7)	
H18A	1.0928	0.7709	0.4240	0.052*	
C19	1.1535 (2)	0.5292 (5)	0.3513 (2)	0.0448 (7)	
H19A	1.2271	0.5925	0.3518	0.054*	
C20	1.1274 (2)	0.3266 (5)	0.30572 (19)	0.0422 (7)	
C21	1.0239 (2)	0.2270 (5)	0.31015 (18)	0.0396 (6)	
H21A	1.0083	0.0867	0.2825	0.048*	
C22	0.9399 (2)	0.3308 (5)	0.35548 (18)	0.0370 (6)	
C23	0.8319 (2)	0.2348 (5)	0.35925 (18)	0.0386 (6)	
H23A	0.8163	0.0910	0.3349	0.046*	
C24	0.7492 (2)	0.3453 (5)	0.39745 (18)	0.0377 (6)	
H24A	0.6773	0.2766	0.3998	0.045*	
C25	1.1868 (3)	0.0424 (6)	0.2127 (2)	0.0524 (8)	
H25A	1.2504	0.0054	0.1794	0.079*	
H25B	1.1171	0.0592	0.1716	0.079*	
H25C	1.1765	−0.0771	0.2548	0.079*	

### Atomic displacement parameters (Å^2^)

**Table:**

	*U*^11^	*U*^22^	*U*^33^	*U*^12^	*U*^13^	*U*^23^
O1	0.0389 (10)	0.0518 (12)	0.0400 (11)	−0.0032 (9)	0.0080 (8)	−0.0110 (9)
O2	0.0376 (10)	0.0382 (11)	0.0349 (10)	−0.0034 (9)	0.0014 (8)	0.0028 (9)
O3	0.0458 (11)	0.0428 (12)	0.0533 (13)	−0.0078 (11)	−0.0001 (9)	0.0072 (11)
O4	0.0406 (11)	0.0560 (14)	0.0512 (13)	0.0009 (10)	0.0087 (9)	0.0020 (11)
C1	0.0360 (14)	0.0447 (16)	0.0323 (14)	−0.0030 (13)	0.0021 (11)	0.0009 (13)
C2	0.0401 (15)	0.0320 (14)	0.0378 (14)	−0.0003 (12)	0.0022 (11)	−0.0012 (12)
C3	0.0420 (16)	0.0466 (17)	0.0394 (16)	−0.0026 (14)	0.0008 (12)	−0.0077 (14)
C4	0.0418 (16)	0.0510 (18)	0.0348 (16)	0.0032 (13)	−0.0010 (12)	−0.0006 (14)
C5	0.0477 (16)	0.0373 (16)	0.0366 (15)	−0.0004 (13)	0.0045 (12)	0.0007 (13)
C6	0.0425 (15)	0.0393 (16)	0.0400 (15)	0.0031 (13)	0.0042 (12)	−0.0012 (13)
C7	0.0386 (14)	0.0395 (16)	0.0347 (14)	−0.0009 (12)	0.0027 (12)	−0.0024 (13)
C8	0.0391 (15)	0.0453 (17)	0.0459 (17)	−0.0037 (14)	0.0078 (12)	−0.0055 (15)
C9	0.0491 (18)	0.0473 (18)	0.0562 (19)	0.0028 (15)	−0.0004 (15)	−0.0025 (16)
C10	0.0509 (18)	0.068 (2)	0.0508 (19)	0.0006 (17)	0.0142 (15)	−0.0085 (18)
C11	0.0463 (17)	0.0502 (19)	0.056 (2)	−0.0064 (15)	0.0090 (14)	−0.0006 (16)
C12	0.0388 (15)	0.0354 (15)	0.0359 (15)	0.0021 (12)	0.0076 (11)	0.0010 (12)
C13	0.0455 (16)	0.0323 (15)	0.0343 (14)	−0.0020 (12)	0.0037 (12)	−0.0028 (12)
C14	0.0584 (18)	0.0465 (18)	0.0372 (16)	0.0009 (16)	0.0053 (13)	0.0014 (15)
C15	0.0395 (14)	0.0357 (15)	0.0309 (14)	−0.0003 (13)	−0.0034 (11)	0.0043 (13)
C16	0.0446 (16)	0.0323 (14)	0.0364 (15)	−0.0023 (12)	−0.0039 (12)	0.0015 (12)
C17	0.0400 (14)	0.0367 (15)	0.0343 (14)	−0.0034 (12)	−0.0027 (11)	0.0062 (13)
C18	0.0436 (16)	0.0407 (16)	0.0442 (17)	−0.0060 (13)	−0.0055 (13)	0.0050 (13)
C19	0.0372 (15)	0.0489 (18)	0.0472 (17)	−0.0064 (14)	0.0005 (13)	0.0099 (15)
C20	0.0379 (15)	0.0473 (18)	0.0410 (16)	0.0021 (13)	0.0026 (12)	0.0070 (14)
C21	0.0419 (16)	0.0388 (16)	0.0371 (15)	−0.0002 (13)	0.0007 (12)	−0.0001 (13)
C22	0.0390 (14)	0.0372 (15)	0.0332 (14)	−0.0001 (12)	−0.0025 (11)	0.0022 (12)
C23	0.0402 (15)	0.0346 (15)	0.0394 (15)	−0.0034 (12)	−0.0015 (11)	−0.0006 (13)
C24	0.0356 (13)	0.0381 (15)	0.0382 (15)	−0.0057 (12)	−0.0001 (11)	0.0003 (13)
C25	0.0499 (17)	0.062 (2)	0.0463 (18)	0.0049 (17)	0.0088 (14)	0.0044 (17)

### Geometric parameters (Å, °)

**Table:**

O1—C7	1.417 (3)	C10—H10C	0.9800
O1—C8	1.446 (3)	C11—H11A	0.9800
O2—C12	1.344 (3)	C11—H11B	0.9800
O2—C1	1.459 (3)	C11—H11C	0.9800
O3—C12	1.214 (3)	C12—C13	1.507 (4)
O4—C20	1.365 (3)	C13—C15	1.520 (4)
O4—C25	1.427 (4)	C13—C14	1.535 (4)
C1—C2	1.538 (4)	C13—H13A	1.0000
C1—C6	1.554 (4)	C14—H14A	0.9800
C1—H1A	1.0000	C14—H14B	0.9800
C2—C3	1.513 (4)	C14—H14C	0.9800
C2—C7	1.540 (4)	C15—C16	1.372 (4)
C2—H2A	1.0000	C15—C24	1.419 (4)
C3—C4	1.324 (4)	C16—C17	1.412 (4)
C3—H3	0.9500	C16—H16A	0.9500
C4—C5	1.504 (4)	C17—C22	1.422 (4)
C4—H4	0.9500	C17—C18	1.428 (4)
C5—C7	1.543 (4)	C18—C19	1.363 (4)
C5—C6	1.560 (4)	C18—H18A	0.9500
C5—H5A	1.0000	C19—C20	1.417 (5)
C6—H6A	0.9900	C19—H19A	0.9500
C6—H6B	0.9900	C20—C21	1.371 (4)
C7—H7A	1.0000	C21—C22	1.420 (4)
C8—C10	1.512 (4)	C21—H21A	0.9500
C8—C11	1.518 (5)	C22—C23	1.409 (4)
C8—C9	1.537 (5)	C23—C24	1.367 (4)
C9—H9A	0.9800	C23—H23A	0.9500
C9—H9B	0.9800	C24—H24A	0.9500
C9—H9C	0.9800	C25—H25A	0.9800
C10—H10A	0.9800	C25—H25B	0.9800
C10—H10B	0.9800	C25—H25C	0.9800
			
C7—O1—C8	116.4 (2)	C8—C11—H11A	109.5
C12—O2—C1	116.9 (2)	C8—C11—H11B	109.5
C20—O4—C25	116.6 (2)	H11A—C11—H11B	109.5
O2—C1—C2	110.2 (2)	C8—C11—H11C	109.5
O2—C1—C6	107.5 (2)	H11A—C11—H11C	109.5
C2—C1—C6	103.7 (2)	H11B—C11—H11C	109.5
O2—C1—H1A	111.7	O3—C12—O2	123.4 (3)
C2—C1—H1A	111.7	O3—C12—C13	124.9 (3)
C6—C1—H1A	111.7	O2—C12—C13	111.7 (2)
C3—C2—C1	106.3 (2)	C12—C13—C15	107.9 (2)
C3—C2—C7	100.5 (2)	C12—C13—C14	110.6 (2)
C1—C2—C7	99.5 (2)	C15—C13—C14	112.2 (2)
C3—C2—H2A	116.0	C12—C13—H13A	108.7
C1—C2—H2A	116.0	C15—C13—H13A	108.7
C7—C2—H2A	116.0	C14—C13—H13A	108.7
C4—C3—C2	107.2 (3)	C13—C14—H14A	109.5
C4—C3—H3	126.4	C13—C14—H14B	109.5
C2—C3—H3	126.4	H14A—C14—H14B	109.5
C3—C4—C5	108.5 (3)	C13—C14—H14C	109.5
C3—C4—H4	125.7	H14A—C14—H14C	109.5
C5—C4—H4	125.7	H14B—C14—H14C	109.5
C4—C5—C7	100.4 (2)	C16—C15—C24	118.7 (3)
C4—C5—C6	106.4 (2)	C16—C15—C13	122.8 (3)
C7—C5—C6	98.9 (2)	C24—C15—C13	118.5 (2)
C4—C5—H5A	116.2	C15—C16—C17	121.6 (3)
C7—C5—H5A	116.2	C15—C16—H16A	119.2
C6—C5—H5A	116.2	C17—C16—H16A	119.2
C1—C6—C5	102.3 (2)	C16—C17—C22	119.1 (2)
C1—C6—H6A	111.3	C16—C17—C18	122.8 (3)
C5—C6—H6A	111.3	C22—C17—C18	118.1 (3)
C1—C6—H6B	111.3	C19—C18—C17	121.0 (3)
C5—C6—H6B	111.3	C19—C18—H18A	119.5
H6A—C6—H6B	109.2	C17—C18—H18A	119.5
O1—C7—C2	113.1 (2)	C18—C19—C20	120.7 (3)
O1—C7—C5	115.4 (2)	C18—C19—H19A	119.6
C2—C7—C5	93.8 (2)	C20—C19—H19A	119.6
O1—C7—H7A	111.2	O4—C20—C21	125.0 (3)
C2—C7—H7A	111.2	O4—C20—C19	115.3 (3)
C5—C7—H7A	111.2	C21—C20—C19	119.7 (3)
O1—C8—C10	103.8 (2)	C20—C21—C22	120.8 (3)
O1—C8—C11	110.9 (3)	C20—C21—H21A	119.6
C10—C8—C11	110.8 (3)	C22—C21—H21A	119.6
O1—C8—C9	109.5 (3)	C23—C22—C21	122.0 (3)
C10—C8—C9	110.1 (3)	C23—C22—C17	118.5 (2)
C11—C8—C9	111.4 (3)	C21—C22—C17	119.4 (2)
C8—C9—H9A	109.5	C24—C23—C22	121.1 (3)
C8—C9—H9B	109.5	C24—C23—H23A	119.5
H9A—C9—H9B	109.5	C22—C23—H23A	119.5
C8—C9—H9C	109.5	C23—C24—C15	120.9 (2)
H9A—C9—H9C	109.5	C23—C24—H24A	119.6
H9B—C9—H9C	109.5	C15—C24—H24A	119.6
C8—C10—H10A	109.5	O4—C25—H25A	109.5
C8—C10—H10B	109.5	O4—C25—H25B	109.5
H10A—C10—H10B	109.5	H25A—C25—H25B	109.5
C8—C10—H10C	109.5	O4—C25—H25C	109.5
H10A—C10—H10C	109.5	H25A—C25—H25C	109.5
H10B—C10—H10C	109.5	H25B—C25—H25C	109.5
